# Cross-cultural adaptation and psychometric validation of the Chichewa (Malawi) PedsQL^™^ 4.0 Generic Core Scales child self-report and PedsQL^™^ 4.0 GCS teen self-report

**DOI:** 10.1186/s41687-024-00761-5

**Published:** 2024-09-10

**Authors:** Lucky Gift Ngwira, Hendramoorthy Maheswaran, Stavros Petrou, Louis W. Niessen, Sarah C. Smith

**Affiliations:** 1 Health Economics and Policy Unit, Kamuzu University of Health Sciences, P/ B 360, Chichiri, Blantyre 3, Malawi; 2 Institute of Global Health Innovation, Imperial College London, London, UK; 3 Nuffield Department of Primary Care Health Sciences, University of Oxford, Oxford, UK; 4 John Hopkins School of Public Health, Baltimore, MD USA; 5 Department of International Health, Liverpool School of Tropical Medicine, Liverpool, UK; 6 London School of Hygiene & Tropical Medicine, London, UK

**Keywords:** PedsQL, HRQoL, Cross-cultural adaptation, ‘Southern Africa’

## Abstract

**Background:**

The PedsQL™ 4.0 Generic Core Scales (GSC) have been translated into over 60 languages, but use in the sub-Saharan African region is limited. This study aimed to cross-culturally adapt and validate the PedsQL™ 4.0 GCS child self-report and teen self-report versions into the Chichewa language for Malawi.

**Methods:**

The English (USA) versions were adapted (translation, back translation and cognitive interviews to evaluate conceptual equivalence) into Chichewa. We recruited 289 children (8–17 years) in Blantyre, Malawi. Classical psychometrics at the item level (missing data, endorsement frequencies, item redundancy) and scale level (internal consistency, convergent, discriminant and known groups validity) was used to evaluate the new Chichewa versions.

**Results:**

Six items were found to need cultural adaptation for Malawi. There were problems with missing data (< 5%) and adjacent endorsement frequency (< 10%) among younger children. Internal consistency reliability was acceptable (Cronbach α > 0.7). Convergent validity was generally strong (correlations > 0.4). Discriminant validity (*p* > 0.05) was evident with respect to gender and age, but not for school grade (*p* < 0.05). Effect sizes indicating known groups validity were in the expected direction but of variable magnitude.

**Conclusion:**

We have successfully adapted the PedsQL™ 4.0 GCS child self-report and teen self-report into Chichewa for use in Malawi. Many aspects of the psychometric evaluation were promising, though some elements were more mixed and we have not yet been able to evaluate test-retest reliability or responsiveness. We suggest that the PedsQL™4.0 GCS child and teen self-reports should be used with caution among children and adolescents in Malawi.

## Introduction

There has been an increase in the development and use of childhood health-related quality of life (HRQoL) measures over the last 30 years in clinical trials, clinical practice, and resource allocation decisions [1, 2]. However, development, validation and use of such instruments in low- and middle- income countries (LMICs) has been limited [3]. The continued decrease in mortality in children and adolescents in some LMICs, particularly in the sub-Saharan African (sSA) region, means that there is likely to be a shift to measuring health outcomes in terms of quality of life (QoL) and HRQoL. Effective measurement of outcomes such as HRQoL is important for establishing burden of disease and for evaluation of health care programmes [4]. The Pediatric Quality of Life Generic (PedsQL)™ 4.0 Generic Core Scales (GCS) self-report is a generic instrument developed for use with healthy, acutely and chronically ill pediatric populations [5]. The PedsQL™ 4.0 GCS assesses HRQoL across four dimensions: physical, emotional, social and school functioning [6].

PedsQL™4.0 GCS was originally developed in the US for use with children aged 2 to 18 years. The instrument was developed to be proxy reported across all age groups and in addition there is an interview assisted version (ages 5–7 years) and a self reported version (child-ages 8–12 years and teen-ages 13–18 years) [5]. The PedsQL™4.0 GCS has demonstrated feasibility, reliability, validity, sensitivity, and responsiveness in healthy and ill children and adolescents [5–7]. Although the PedsQL™ 4.0 GCS has been translated into over 60 languages [7], its use in the sSA region has been limited [3]. This study aimed to cross-culturally adapt and validate the PedsQL™ 4.0 GCS (child self-report and teen self-report versions) into Chichewa, Malawi’s national language.

## Methods

### The instruments: the Pediatric Quality of Life ^TM^ version 4.0 Generic Core Scales

The PedsQL ^TM^ 4.0 GCS child self-report and teen self-report versions were provided by the Mapi Research Trust [8]. With exception of the use of ‘kids’ and ‘teens’, respectively, in some items, the content is the same for the two instruments. Both versions of the instrument have 23 items and each is reported on a 5-point Likert type scale (never a problem; almost never a problem; sometimes a problem; often a problem; almost always a problem). The PedsQL ^TM^ 4.0 GCS generates a total scale score for the 23 items; two summary scores: Physical Functioning summary score (8 items) and Psychosocial summary score (15 items); and sub-scale scores for each of 4 sub-scales: physical functioning (8 items), emotional functioning (5 items), social functioning (5 items), and school functioning (5 items). Scores are not computed if less than 50% of the relevant items are complete. For respondents with at least 50% items complete, missing data are imputed with the mean of completed items. Each score is then obtained by reversing the constituent items and linearly transforming each item to 0–100, summing the relevant items and dividing by the number of items answered to give a mean score ranging from 0–100 [5, 9]. Higher scores indicate better HRQoL.

The cross-cultural adaptation and psychometric validation of the Chichewa (Malawi) PedsQL ^TM^ 4.0 GCS child and teen self-report versions took a two step process: first, cross cultural adaptation of the instrument and second, psychometric validation.

Step1: Establishing linguistic and conceptual equivalence between the original PedsQL ^TM^ 4.0 GCS child self-report and teen self-report and the Malawi (Chichewa) version.

#### Translation process

Alongside the instruments, the Mapi Research Trust also provided us with a standard PedsQL ^TM^ 4.0 GCS translation protocol (forward and backward), and cognitive interview protocols to establish linguistic and conceptual equivalence [8]. Following these protocols, two experienced local translators independently translated the PedsQL ^TM^ 4.0 GCS from English (US) into Chichewa. A second pair of translators whose first language was English, but were also fluent in Chichewa, independently back translated from Chichewa into English. Any inconsistencies, including addition and removal of some text, were resolved through discussion between the translators and the first author (who was fluent in both Chichewa and English) through consensus. In order to establish conceptual equivalence, the agreed versions then went through a cognitive interview process.

#### Cognitive interviews

Ten healthy participants (five for each age version) who had consented to taking part in cognitive interviews took part in this exercise. First, participants self-completed the translated PedsQL ^TM^ 4.0 GCS questionnaire in a one to one setting. Help to understand the instructions was given and recorded on a form by the interviewer, but no help was given about how to respond to the questions.

After completing the questionnaires, each participant took part in a one-to-one interview. The participants were asked to explain why they had given their answers to each question. In addition, participants were asked to say what they thought might have been missing in the questionnaire, how to improve the wording, and to give specific examples to improve response levels. This was done to understand how each item was understood by respondents and to check that the local understanding matched what was intended by the developers. For any items that were identified to be understood differently from what was intended, as well as for any given suggestions, respondents were asked to suggest alternative wording in the interviews. These suggestions were written down on a data collection form by the interviewer capturing the alternative wording. In an iterative fashion, the suggested wording was re-checked in interviews with the same participants as well as in subsequent interviews with different participants to ascertain the most appropriate wording. The final wording that was used for the pre-final versions was proofread for typographical and layout errors. The pre-final versions were then forwarded to the Mapi Research Trust for approval following which they were administered to children and adolescents as outlined below.

##### Step 2: self-completion of the questionnaire

Once consent was obtained as outlined below, the translated and adapted questionnaires were distributed by the research team on a school day in a classroom setting or at the end of clinical care in a hospital setting. After completing the questionnaires, these were handed over and collected by the study staff. Only children (*n* = 289) who were literate were included, but critically ill children were excluded from recruitment.

### Participants and recruitment process

The sample for the cognitive interviews were recruited from healthy participants only from one primary school in Blantyre, Malawi, and none of the participants were already familiar with the PedsQL ^TM^ 4.0 GCS. A convenience sample of healthy children (8–12 years) and adolescents (13–17 years) were recruited. Invitations to participate in the study were made through the school via a teacher. Participants took the study information leaflets and consent forms home for receipt of consent by their respective parents/guardians and these were brought back to the school the following day. Subsequently a date was set up for the cognitive interviews in the school setting. Only children and adolescents that had both written assent and consent from themselves and their respective parents/guardians took part in the interviews.

The psychometric evaluation sample similarly consisted of a convenience sample of healthy and sick children (8–12 years) and adolescents (13–17 years) in Blantyre, Malawi. Healthy participants were recruited from five local schools and sick participants from an outpatient department at the Queen Elizabeth Central Hospital, the main referral hospital in Malawi. A similar approach for obtaining written assent and consent for healthy participants as for the cognitive interviews was employed for the psychometric evaluation. On the otherhand, written assent and consent for sick participant was only obtained from children and their parents/guardians at the end of their clinical care. Only children who were able to self-complete the questionnaires, as evident from written consenting process, were included. In addition, critically ill children were excluded from recruitment.

Ethical approval for this study was granted by Ethics Committees at the then Malawi College of Medicine (now KUHeS) (P.10/18/2509) and Liverpool School of Tropical Medicine (19–045). Although psychometric evaluation often relies on correlations and does not necessarily require a power calculation, to show differences in the known-groups validity, a sample size of 200 participants was calculated to provide 80% power, at the two-sided significance level of 0.05.

### Psychometric analyses

Data analysis was performed using IBM SPSS 26.0.0 for Mac (IBM Corp. Armonk, New York, USA) [10]. Analyses were undertaken separately for the two age groups: child (8–12 years) and teen (13–17 years) scales.

#### Item analysis

We undertook item analysis to understand the contribution of individual items within each scale using the following criteria.

Missing data: A criterion of 5% was used to evaluate missing data [11]. Items that had ≥ 5% missing data were flagged as potentially problematic.

Maximum endorsement frequency: This is the endorsement at the extremes of the response scale. Items were considered problematic if they had > 80% endorsement at either end of the scale [12]. This indicated whether responses were concentrated at the top or bottom end of the response scale (floor or ceiling effect).

Aggregate adjacent endorsement frequency: This is the extent to which adjacent response options sum to at least a specified minimum [13]. Items were considered problematic if any two or more adjacent response options summed to < 10% [13]. This provided information as to whether there were some response options that were not being used.

Item redundancy: This reflects the extent to which each item within the scale made a unique contribution and was not over-lapping with other items in the scale. It was assessed by evaluating the inter-item correlation between all items in the scale. In this study, items with inter-item correlations > 0.75 were considered problematic [12].

#### Reliability

Reliability indicates the extent to which a scale is free from random error and therefore able to produce consistent and reproducible results [12]. We set a criterion that the Cronbach’s alpha should be ≥0.70 [14].

#### Validity

##### Convergent validity

This is the extent to which similar dimensions of two or more instruments are related and therefore expected to have a moderate to strong correlation. It was hypothesized that there would be moderate to strong between sub-scale correlations (> 0.4) for both PedsQL ^TM^ 4.0 GCS child self-report and teen self-report. In addition, we also evaluated the correlation of the Chichewa versions of the PedsQL ^TM^ 4.0 sub-scales and EQ-5D-Y-3L and EQ-5D-Y-5L dimensions but these results have been published elsewhere [15].

##### Discriminant validity

Discriminant validity is the extent to which an instrument is not correlated with a measure of something unrelated. It was anticipated that gender, age and education (school grade) would not be factors that determine self-completion of the scales. It was therefore hypothesized that there would be no association between PedsQL™ 4.0 GCS total scores with gender, age or grade. We expected no difference in PedsQL™ 4.0 GCS scores by gender (non-significant *t*-test). It was also anticipated that there would be correlation < 0.2 between PedsQL™ 4.0 GCS self-report sum scores and age. To investigate the relationship with grade, we categorised this variable into three categories based on the general distribution of children (who were attending primary and secondary school) and in line with the former scaling for primary school education in Malawi. Grades 1–5 formed a lower primary category (group 1), grades 6–8 an upper primary category (group 2), and grades 9–12 formed a secondary/high school category (group 3). We investigated the relationship between these using ANOVA and expected a non-significant relationship.

##### Known-groups validity

Known-group validity is the extent to which scores differ for two or more groups that are known to be different in some other aspects e.g., health status. It was hypothesized, based on previous findings, [5] that PedsQL™ 4.0 GCS sum scores would be worse for sick compared with healthy children. Due to the small number of children recruited with a chronic condition, this group was combined with the acute group to form a single sick group. The relationship between healthy and sick participants was investigated using a *t*-test and the magnitude of the difference was assessed using effect sizes. Cohen’s criterion for effect sizes was used: <0.2 poor, 0.3–0.49 small, 0.5–0.8 moderate, and > 0.8 large [11, 16]. It was further hypothesized that the effect size magnitude between the healthy and sick groups would be moderate to large.

##### Item convergent/discriminant (within scale) validity

We further investigated the role of each item within its scale using item convergent and discriminant validity; each item should be more highly associated with its own sub-scale (within) than with other sub-scales (between) [17]. To quantify the difference between the within sub-scale and between sub-scale correlations, we adapted the multitrait-multimethod (MTMM) approach developed by Campbell and Fiske [18] but modified by Henseler et al. [19]. The ratio was calculated using Microsoft excel as the average of between scale correlations relative to the average of within scale corrections [19]. It was hypothesized that all the ratios would be below the 0.85 (conservative approach) threshold to support evidence for within sub-scale validity [19].

## Results

### Participants

Each of the cognitive interviews consisted of five healthy children (mean 11 years) and adolescents (mean 13 years) for the PedsQL™ 4.0 GCS child and teen version respectively.

For the psychometric evaluation, a total of 289 participants completed the PedsQL™ 4.0 GCS aged 8–17 years (mean 13.6, median 14) (Table 1). Of these, 191 completed the PedsQL™4.0 GCS teen self-report, and 98 completed the PedsQL™ 4.0 GCS child self-report. There were slightly more female participants (56%) compared to males, and slightly more (55%) in primary school (lower and upper) compared to those in secondary school. Most participants were healthy (67%) in comparison to those that were sick.

**Table 1:** Participant characteristics for the PedsQL™ 4.0 GCS child self-report and teen self-report

Characteristic	*N* (%)	PedsQL 4.0 child self-report	PedsQL 4.0 teen self-report
Participants	289	98	191
Gender*			
Male	121 (44%)	39	82
Female	153 (56%)	51	102
Age in years^#^			
8–12	96 (34%)	85	11
13–17	185 (66%)	8	177
Health condition			
Healthy	95 (33%)	12	83
Acute	155 (54%)	85	70
Chronic	39 (13%)	1	38
School grade**			
Group 1	71 (25%)	53	18
Group 2	97 (35%)	40	57
Group 3	111 (40%)	0	111

*missing data: 15 (child self-report = 8, teen self-report = 7)

^#^missing data: 8 (child self-report = 5, teen self-report = 3)

**group 1:grades 1–5, group 2:grades 6–8, group 3: grades 9–12

### Cross-cultural adaptation: linguistic and conceptual equivalence

#### Translation process

The translation process identified five items that did not directly translate into Chichewa. For example, ‘It is hard for me to walk more than one ***block’*** was difficult to conceptualize since local distance is not measured in blocks in a Malawian setting. Distance was therefore conceptualized, by the translation team but not tested with participants, in terms of classrooms, for children to easily relate to, where one block would be equivalent to three double classrooms. The item about ‘sports activity or exercise’, was also difficult to translate since sports and exercise have one word in Chichewa, ‘*masewero*’. As such both English words had the same Chichewa rendering. A similar approach was taken for ‘taking shower or bath’ which have the same word in Chichewa, ‘*kusamba’*. While ‘*kupweteka’* is a correct translation for ‘hurt’ as well as ‘aching’—but in this age group, ‘*kuwawa’* was deemed more appropriate. As indicated in the section “Methods”, for all of these issues, some texts were either added or removed accordingly to come up with a more accurate translation. Additionally, aches in Chichewa is a noun and needs a supporting verb which isn’t there so this was added. Lastly, ‘Not feeling well’ was translated as ‘kusapeza bwino’ and ‘kudwala’ but the former can also be used to imply lack of things i.e., poverty. For this reason, ‘kudwala’ was retained as a more fitting translation for not feeling well.

#### Cognitive interviews

The cognitive interviews revealed one major conceptual issue that needed revision. The item “It is hard for me to take a bath or shower by myself” was translated “Zikumandivuta kusamba”. However, it was discovered during cognitive interviews that ‘kusamba’ has a cultural connotation to ‘menstrual cycle’ for adolescent girls. Consequently, “m’thupi” (body) was added to the translation to read “Zikumandivuta kusamba m’thupi”. This resolved any errors to the meaning and no additional changes were suggested by the participants.

The cognitive interviews generally showed that participants understood the questionnaires as they gave specific, appropriate examples for response levels. However, self-completion of the questionnaires without interviewer assistance was problematic in this setting. For example, some participants would circle every box instead of choosing one response per item. Once the interviewer clearly explained the instructions to the participants, this addressed the problem, and versions were proofread and forwarded to the Mapi Trust for official approval.

### Psychometric analysis

#### Item analyses

In general, missing data (> 5%) was high amongst the younger children; 16 of 23 items failed the criterion (Table 2). Amongst the adolescents, missing data was not so much of a problem as only 1 of 23 items failed the criterion. There were no problems with maximum aggregate endorsement frequency. Most items showed problems with aggregate adjacent endorsement (20 out of 23 items in both versions of the instrument). No items failed the item redundancy criterion for either the child or teen version of the PedsQL 4.0 GCS.

**Table 2:** Item-level analysis for PedsQL^™^ 4.0 child self-report and PedsQL^™^ 4.0 teen self-report*

PedsQL™ 4.0			Missing data		Maximum endorsement frequency		Adjacent aggregate endorsement frequency		Item redundancy	
Sub-scales	Items		Child	Teen			Child	Teen		
Physical Functioning	1.1	To walk more than one block	x				x	x		
	1.2	To run					x	x		
	1.3	Sports activity or exercise						x		
	1.4	To lift something heavy	x							
	1.5	To take a bath or shower by myself	x				x	x		
	1.6	To do chores around the house					x	x		
	1.7	Hurt or ache					x	x		
	1.8	Low energy	x				x	x		
Emotional Functioning	2.1	Afraid or scared					x			
	2.2	Sad or blue					x	x		
	2.3	Angry	x				x	x		
	2.4	Trouble sleeping	x				x	x		
	2.5	Worry about what will happen to me	x				x	x		
Social Functioning	3.1	Trouble getting along with other children	x				x	x		
	3.2	Other children do not want to be my friend	x				x	x		
	3.3	Other children tease me	x				x	x		
	3.4	Cannot do things that other children can do	x				x	x		
	3.5	Keep up when I play with other children	x					x		
School Functioning	4.1	Pay attention in class					x	x		
	4.2	Forget things	x	x			x			
	4.3	Trouble keeping up with my schoolwork	x				x	x		
	4.4	Miss school because of not feeling well	x				x	x		
	4.5	Miss school to go to doctor or hospital					x	x		

*no item failed for maximum endorsement frequency and item redundancy; x indicates a fail on a criteria

#### Reliability

The reliability findings as calculated by Cronbach’s alpha coefficients for the PedsQL™ 4.0 child self-report and PedsQL™ 4.0 teen self-report were > 0.7 threshold. All the Cronbach’s alpha values for sub-scales and summary scores ranged between 0.84 and 0.94, and the overall total score was > 0.94.

#### Validity

##### Convergent validity

The between sub-scale correlation coefficients were above 0.40 for all of PedsQL™ 4.0 GCS child self-report sub-scales to support evidence of convergent validity: Physical Functioning 0.40–0.74, Emotional Functioning 0.41–0.70, Social Functioning 0.42–0.66, and School Functioning 0.40–0.74. The PedsQL™ 4.0 GCS teen self-report similarly had most of the between sub-scale correlation coefficients above 0.40: Physical Functioning 0.39–0.79, Emotional Functioning 0.39–0.67, Social Functioning 0.48–0.72, and School Functioning 0.49–0.87.

##### Discriminant validity

There was evidence to support the discriminant validity of both PedsQL™ 4.0 GCS child self-report and PedsQL™ 4.0 GCS teen self-report. There was no significant difference by gender (Table 3) for the total score (child total score mean difference = 6.26, *t* = 1.716, *p* < 0.900; teen total score mean difference = 2.71, *t* = 1.062, *p* < 0.290). Most of the sub-scales also showed no significant differences by gender. The exception was the Social Functioning sub-scale for the PedsQL™ 4.0 GCS child self-report (mean difference = 11.23, *t* = 2.761, *p* < 0.007).

**Table 3:** Discriminant validity by gender for PedsQL™ 4.0 child self-report (8–12 years) and teen self-report (13–18 years)

Scale	PedsQL 4.0 child self-report (*n* = 98, male = 38, female = 50)			PedsQL 4.0 teen self-report (*n* = 182, male = 81, female = 101)		
	mean difference	*t*-test		mean difference	*t*-test	
		t-statistic*	*p*-value		t-statistic*	*p*-value
Total Scale score	6.26	1.716	0.090	2.71	1.062	0.290
Psychosocial Summary Health	7.11	1.937	0.056	2.86	1.077	0.283
Physical Summary Health	2.18	0.481	0.632	1.90	0.715	0.477
Emotional Functioning subscale	3.41	0.762	0.448	2.52	0.911	0.364
Social Functioning subscale	11.23	2.761	**0.007**	5.20	1.748	0.082
School Functioning subscale	5.40	1.269	0.208	1.89	0.571	0.569

Bold indicates statistical significance

*assume equal variance

There was also support for discriminant validity in terms of age. The correlation between age and PedsQL™ 4.0 GCS child self-report total, summary and sub-scale scores ranged from 0.10 to 0.15. Similarly, the correlation between age and PedsQL™ 4.0 GCS teen self-report scores ranged between 0.01 and 0.16. All values were in the acceptable range.

Discriminant validity was also evident in relation to grade (Table 4) for child report (non-significant difference by school grade for total, summary physical, summary psychosocial, and all sub-scales).

**Table 4:** PedsQL™ 4.0 child self-report and teen self-report discriminant validity by grade

Sub-scale		PedsQL 4.0 child self-report^#^				PedsQL 4.0 teen self-report^$^			
		(grade* group1 = 53, group2 = 40)				(grade* group1 = 18, group2 = 57, group3 = 111)			
		df	Mean Square	F	Sig.	df	Mean Square	F	Sig.
Physical Functioning sub-scale	Between Groups	1	15.6	0.035	0.851	2	1753.2	5.735	**0.004**
	Within Groups	89	441.2			181	305.7		
	Total	90				183			
Emotional Functioning sub-scale	Between Groups	1	518.3	1.248	0.267	2	318.5	0.942	0.392
	Within Groups	87	415.3			176	338.1		
	Total	88				178			
Social Functioning sub-scale	Between Groups	1	239.6	0.654	0.421	2	1981.2	5.231	**0.006**
	Within Groups	84	366.3			179	378.7		
	Total	85				181			
School Functioning sub-scale	Between Groups	1	1079.3	2.935	0.090	2	1851.0	3.969	**0.021**
	Within Groups	86	367.8			176	466.3		
	Total	87				178			
Psychosocial Health Summary	Between Groups	1	633.3	2.296	0.133	2	1157.3	3.882	**0.022**
	Within Groups	84	275.8			174	298.1		
	Total	85				176			
Physical Health Summary	Between Groups	1	15.6	0.035	0.851	2	1753.2	5.735	**0.004**
	Within Groups	89	441.2			181	305.7		
	Total	90				183			
Total Scale Score	Between Groups	1	292.0	1.078	0.302	2	1504.4	5.600	**0.004**
	Within Groups	83	270.8			173	268.6		
	Total	84				175			

Bold indicates statistical significance

^#^missing data = 8

^$^missing data = 2

*grade group1 = grade 1–5; group2 = grade 6–8; group3 = grade 9–12

For the PedsQL™ GCS 4.0 teenself-report, however, there was a significant mean difference for total, summary physical, summary psychosocial, and all sub-scales except emotional functioning by school grades.

##### Known-groups validity

Both the child self-report and teen self-report overall score effect sizes indicated reasonable known-groups validity between the healthy and sick children; between group differences were in the expected direction (worse scores for sick children) but effect sizes were variable. For the PedsQL™ 4.0 GCS teen self-report, the effect size ranged from small (0.21 for social functioning) to large (0.93 physical functioning) for the sub-scale scores. Generally, the PedsQL™ 4.0 GCS child self-report displayed small effect sizes for all the sub-scale scores and total scale score (Table 5).

**Table 5:** PedsQL™4.0 child self-report (8–12 years) and teen self-report (13–18 years) known-group validity

Scale	PedsQL 4.0 child self-report (*N* = 98, healthy = 12, 81 = sick)					PedsQL 4.0 teen self-report (*N* = 191, healthy = 83, 106 = sick)				
	t-statistic^#^					t-statistic^#^				
	t	p-value	MD	SD	Effect size *	t	p-value	MD	SD	Effect size*
Total Scale Score	0.751	0.455	4.14	20.75	0.20	3.720	0.000	9.06	11.41	0.79
	0.634	0.538	4.14	16.62		3.915	0.000	9.06	19.37	
Physical Health Summary	0.914	0.363	6.31	19.70	0.32	4.271	0.000	10.76	11.59	0.93
	0.987	0.341	6.31	21.74		4.550	0.000	10.76	20.52	
Psychosocial Health Summary	0.578	0.565	3.08	20.81	0.15	3.374	0.001	8.63	13.07	0.66
	0.490	0.632	3.08	16.66		3.524	0.001	8.63	19.82	
School Functioning sub-scale	0.700	0.485	4.22	22.04	0.19	3.712	0.000	11.69	15.29	0.76
	0.629	0.540	4.22	19.09		3.919	0.000	11.69	24.89	
Social Functioning sub-scale	0.381	0.704	2.34	19.36	0.12	3.932	0.000	11.13	15.04	0.74
	0.388	0.703	2.34	19.86		4.097	0.000	11.13	22.00	
Emotional Functioning sub-scale	0.394	0.694	2.55	22.05	0.12	1.237	0.218	3.38	16.42	0.21
	0.377	0.712	2.55	20.76		1.262	0.208	3.38	19.88	
Physical Functioning sub-scale	0.914	0.363	6.31	19.70	0.32	4.271	0.000	10.76	11.59	0.93
	0.987	0.341	6.31	21.74		4.550	0.000	10.76	20.52	

*MD* mean difference, *SD* standard deviation

^#^assuming equal variance

*effect size designated as < 0.2 poor, 0.3–0.49 small, 0.5–0.8 moderate, and > 0.8 large

##### Item convergent/discriminant (within scale) validity

The MTMM matrix results in Table 6 shows that the within scale validity criterion was met. The sub-scale inter-item relationship ratios were all within the criterion threshold of < 0.85 as hypothesized. However, for some of the sub-scales, the ratio was very close to the set threshold value. For example, among the children who self-completed the PedsQL™4.0 GCS child self-report, the highest MTMM ratio (0.84) was in the Social Functioning / School Functioning matrix. Among adolescents who completed the PedsQL™4.0 GCS child teen-report, the highest matrix ratio (0.84) was the Physical Functioning / School Functioning ratio. These high ratios would indicate that even though the criteria for within scale validity was met evidence for discriminant validity could not be completely ruled out.

**Table 6:** Item convergent/ discriminant validity for PedsQL™4.0 child self-report and teen self-report by sub-scale

Sub-scale	PedsQL 4.0 child self-report				PedsQL 4.0 teen self-report			
	Physical Functioning	Emotional Functioning	Social Functioning	School Functioning	Physical Functioning	Emotional Functioning	Social Functioning	School Functioning
Physical Functioning	–				–			
Emotional Functioning	0.761	–			0.785	–		
Social Functioning	0.579	0.711	-		0.710	0.760	–	
School Functioning	0.541	0.606	0.841	–	0.843	0.774	0.755	–

## Discussion

After making slight adaptations to six items, linguistic and conceptual equivalence was established between the PedsQL ^TM^ 4.0 GCS child self-report and the PedsQL ^TM^ 4.0 GCS teen self-report in Chichewa for Malawi and the original US English versions. The psychometric validation showed that the Chichewa (Malawi) PedsQL™ 4.0 GCS child self-report and teen self-report demonstrated mixed psychometric results across age groups. This suggests that both instruments should be used with some caution among children and adolescents in Malawi.

Overall, the findings showed the value of including the cognitive interviews to establish both linguistic and conceptual equivalence as opposed to only translation. The issue of ‘kusamba’ meaning menstrual cycle was fundamental and this would not have been identified with translation alone. However, although the cognitive interviews identified aspects of the PedsQL ™4.0 GCS concept of health that were not understood in the same way in Chichewa, it did not identify aspects of health that are relevant in Chichewa but are missing from the instrument. Further work to develop a conceptual framework capturing aspects of health relevant to children and adolescents in Malawi is being prepared for publication elsewhere [20].

The relatively high level of missing data for the PedsQL™4.0 GCS child self-report suggests that self-completion may be more challenging for younger children as observed elsewhere [6, 21]. However, the missing data across all the age groups in the analysis based on health status (healthy versus sick) suggest that the missing data may reflect characteristics of the content of the instrument rather than the inability of younger children to respond. Difficulties with content are further supported by the large number of items that failed the adjacent aggregate endorsement. There are two possibilities for this, either children found some of the response options not helpful, or the content of the items was not targeted accurately to match their experience. Other studies have also observed this problem when health outcome instruments are utilised in a healthy population [22]. Further qualitative investigation of the appropriateness and completeness of the items content in PedsQL 4.0 GCS for children in Malawi would therefore be beneficial. This study is one such step to having validated this health outcome instrument for use in these settings. In addition, the circling of all responses by some children unless provided with clear instruction by the interviewer may point to difficulties to self-complete among this age group. It may be necessary to instead use an interviewer-assisted instrument instead of a self-complete one for this age group.

The mixed results of the psychometric evaluation of both versions of the PedsQL™4.0 GCS may also warrant need for further investigation. While the lack of discriminant validity by grade in the PedsQL™ 4.0 GCS teen self-report might have been skewed by a small number of children in the school grade for standards 1–5, the failure to meet criteria for discriminant validity across all age groups in all sub-scales may indicate that school grade is a factor in self-completion of questionnaire. This may well support the notion that a minimum level of education/reading ability is necessary to better understanding the questionnaire.

Limitations of the study need to be considered. First, the PedsQL™4.0 GCS child self-report and teen self-report were conceptually adapted through cognitive interviews with healthy participants only. We did not investigate whether the concepts were understood differently by sick patients, although it is unlikely that this would have changed the translation text. The PedsQL™4.0 GCS child self-report and teen self-report are generic instruments intended to measure HRQoL of both healthy and sick children and therefore there is potential benefit from including both the healthy and those with health conditions in the adaptive process. Secondly, due to limitations imposed by the first COVID-19 restrictions at the time of data collection, data for the psychometric evaluation were collected at one time point only and as a result neither test-retest reliability nor responsiveness could be assessed in this study. Test-retest reliability is necessary to establish if the instrument is stable over time and responsiveness ensures that the instrument detects meaningful changes over time. These are important psychometric features of an instrument and need to be investigated.

## Conclusion

We have successfully adapted the PedsQL™ 4.0 GCS child self-report and teen self-report into Chichewa for use in Malawi. Many aspects of the psychometric evaluation were promising, though some elements were more mixed and we have not yet been able to evaluate test-retest reliability or responsiveness. We suggest that the PedsQL™4.0 GCS child and teen self-reports should be used with caution among children and adolescents in Malawi.

## Data Availability

The datasets generated and analysed during the current study are not publicly available as consent was not sought from participants for such but are available from the corresponding author on reasonable request.
